# Urban–Rural Disparities in Geographic Healthcare Accessibility: A Comparative Study of Nigeria and Zambia

**DOI:** 10.1007/s11524-026-01077-z

**Published:** 2026-04-16

**Authors:** Weixuan Fang, Wen-Bin Zhang, Dorothea Woods, Heather Chamberlain, Winfred Dotse-Gborgbortsi, Iyanuloluwa Deborah Olowe, Marcello Schiavina, Borys Nosatiuk, Rhorom Priyatikanto, Maksym Bondarenko, Joachim Maes, Lewis Dijkstra, Andrew J. Tatem, Alessandro Sorichetta

**Affiliations:** 1 WorldPop, School of Geography and Environmental Science, University of Southampton, Southampton, SO17 1BJ UK; 2 NTT DATA, Rue de Spa 8, 1000 Bruxelles, Belgium; 3 European Commission, Directorate-General for Regional and Urban Policy, 1049 Brussels, Belgium; 4 Directorate for Fair and Sustainable Economy, European Commission Joint Research Centre (JRC), Ispra, Italy; 5 Dipartimento Di Scienze Della Terra “Ardito Desio”, Università Degli Studi Di Milano, Via Mangiagalli 34, 20133 Milan, Italy

**Keywords:** Degree of Urbanisation, Geographic access, Gridded population data, Sustainable Development Goal 3, Travel time

## Abstract

Geographic access to healthcare remains a critical barrier to health equity in low- and middle-income countries, where infrastructure and service provision are unevenly distributed. Using the UN-endorsed Degree of Urbanisation framework, we assessed disparities in geographic healthcare accessibility across Nigeria and Zambia in 2020. Travel times to health facilities were modeled for both walking and motorized transport at 1 km resolution, and stratified by settlement type and demographic group. Results showed marked urban–rural disparities: while city residents could typically reach hospitals within minutes, rural populations faced journeys exceeding 4 h on foot. Motorized transport substantially improved accessibility but remained unavailable to many, leaving only 8% of Nigeria’s population and 6% of Zambia’s population within a 15-min walking distance of a hospital. Children in rural Nigeria were particularly disadvantaged, with longer hospital travel times compared to reproductive-age women and elderly adults, whereas demographic differences were less pronounced in Zambia. These findings highlight persistent geographic and demographic inequalities in access to care, underscoring the need for targeted investment in rural transport networks, strategic placement of health facilities, and tailored support for vulnerable populations. By linking harmonized urban–rural classifications with demographic health needs, this study provides actionable evidence for advancing universal health coverage and reducing inequities in rapidly urbanizing African contexts.

## Introduction

Equitable access to healthcare is widely recognized as a cornerstone of health equity and universal health coverage (UHC). It is explicitly highlighted in Sustainable Development Goal 3 (SDG3), which aims to “ensure healthy lives and promote well-being for all at all ages” [1]. A large body of research has demonstrated that healthcare access is shaped by multiple dimensions, including affordability, availability, acceptability, and geographic accessibility [2]. Among these, geographic access, i.e., how easily people can reach a health facility, plays a particularly critical role, as physical distance and travel time often determine whether individuals are able to obtain timely and effective care [3, 4]. Long travel times are associated with reduced service utilization, delayed care-seeking, and poorer health outcomes [5], especially in low- and middle-income countries (LMICs) where health systems are under-resourced [6, 7].

Healthcare accessibility is widely recognized as unequal across urban and rural areas, with rural populations often experiencing longer travel times, limited healthcare facilities, and fewer healthcare professionals [8, 9]. Geographical accessibility modeling has been widely used to identify gaps in healthcare access and guide targeted interventions, such as optimizing facility placement and improving transport networks [10–12]. However, comparing geographic healthcare accessibility across countries by urban and rural breakdowns presents challenges due to differing national definitions. Countries define areas as urban based on varying criteria [13], such as population density, administrative boundaries, or infrastructure development, which can lead to inconsistencies in cross-country comparisons [14].

The UN-endorsed Degree of Urbanisation [15] method provides a systematic way to delineate the urban–rural continuum for international and regional statistical comparison purposes, supporting the collection of harmonized indicators requested by global agendas, e.g., the SDGs [16]. This globally harmonized method was explicitly designed to monitor access to services and infrastructure in areas with different population sizes and densities and is more objective in that it does not factor the presence of facilities into the definition [17]. By leveraging geospatial data, the Degree of Urbanisation framework enables policymakers to assess how travel time varies across different settlement classes and population distributions, thereby supporting targeted interventions to improve healthcare access.

Building on recent DEGURBA-based analyses of geographic healthcare accessibility [17], this study extends the literature by introducing a comparative two-country design and an explicit demographic perspective. Specifically, we examine how geographic access to health facilities differs not only across the urban–rural continuum but also among key population groups in Nigeria and Zambia. We apply the Degree of Urbanisation method to analyze and compare geographic healthcare accessibility in these two countries, which exhibit distinct urbanisation patterns and demographic structures, with a focus on variations in travel time across demographic groups, transport modes, and degrees of urbanisation. Geographic access to all types of health facilities is first estimated for each 1 km grid cell using WorldPop’s Global2 population estimates [18], considering both motorized transport and walking. We then analyze population-weighted travel times for cities (urban centers), towns and semi-dense areas (urban clusters), and rural areas, as classified by the Degree of Urbanisation. The distribution of the population across travel times for different urbanization levels was compared to contrast inequalities within urbanization levels, and population shares are summarized using multiple time thresholds. Finally, we assess geographical healthcare accessibility for children, women of reproductive age, and older adults, disaggregated by urbanization level.

By identifying gaps in geographic healthcare accessibility, this study provides evidence-based insights for supporting the planning of health systems and resource allocation in Nigeria and Zambia. The findings contribute to the broader discourse on spatial healthcare equity and the need for data-driven policies that ensure healthcare facilities are equitably distributed across diverse population groups.

## Methods

We examined geographic healthcare accessibility in Nigeria and Zambia for the year 2020. Both countries are in sub-Saharan Africa but differ in population size, demographic structure, and health system organization. Nigeria, Africa’s most populous country, is highly urbanized with regional inequalities, while Zambia has a smaller and more dispersed population. Both countries face challenges in extending equitable health coverage to rural and peri-urban communities.

### Gridded Population Data

We used the WorldPop Global2 gridded population distribution data [18], which provide high-resolution annual estimates of population counts for all countries. These data are produced by disaggregating census counts to 100 m grid cells using a machine learning approach that integrates detailed census age–sex structures with geospatial covariates such as land cover, night-time lights, and settlement extents. For this study, we aggregated the data to 1 km resolution to align with the Degree of Urbanisation classification. We focused on three demographic subgroups with distinct health needs: children (0–4 years), reproductive-age women (15–49 years), and the elderly (65 + years).

### Degree of Urbanisation

Settlements were categorized using the UN-endorsed Degree of Urbanisation (DEGURBA) framework [15]. This method classifies all land into three settlement types based on population density and contiguity:

- Cities (urban centers): contiguous areas with ≥ 1500 residents per km^2^ and a total population ≥ 50,000.
- Towns and semi-dense areas (urban clusters): areas with ≥ 300 residents per km^2^ and population between 5000 and 50,000.
- Rural areas: all remaining low-density areas.

This harmonized approach avoids reliance on administrative boundaries or service presence, enabling consistent cross-country comparison [16, 17]. DEGURBA grids were generated at 1 km resolution using WorldPop data and the GHS-DUG v6.1 tool [19].

### Health Facilities Data

Health facility locations were obtained from GRID3 (Geo-Referenced Infrastructure and Demographic Data for Development), which compiles official national health registries and validated geographic data. For Nigeria, we used the GRID3 NGA Health Facilities v2.0 dataset [20], which integrates the Nigeria Health Facility Registry with GRID3 updates. For Zambia, we used the GRID3 Operational Health Facility Layer [21]. Both datasets include geographic coordinates and facility types, allowing separate analyses of access to any health facility versus hospitals only. The GRID3 facility datasets aim to provide comprehensive national coverage by integrating official health facility registries with geospatial validation and updates from multiple sources. Included facilities comprise public and private health facilities across primary, secondary, and tertiary levels, although coverage and classification accuracy may vary across regions, particularly in remote rural areas where informal or recently established facilities may be under-represented.

### Travel Time Mapping

We estimated least-cost travel times from each 1 km grid cell to the nearest facility using global friction surfaces from the Malaria Atlas Project [7]. These estimates represent optimal free-flow travel conditions and do not capture real-world delays such as traffic congestion, waiting times, or transport availability; however, they provide a consistent and widely used basis for comparative assessments of geographic accessibility across settings. Two scenarios were modeled:

- Motorized transport: assuming consistent access to vehicles, boats, or rail.
- Walking: assuming no access to motorized transport.

Travel times were calculated using the Distance Accumulation tool in ArcGIS Pro v3.3, yielding harmonized cross-national estimates suitable for comparative analysis.

### Statistical Analysis

We calculated average population-weighted travel times to the nearest facility, stratified by DEGURBA class, country, facility type, transport mode, and demographic subgroup. We also summarized the proportion of the population within fixed thresholds of 15, 30, 60, and 120 min of a facility, reflecting common benchmarks used in health equity and emergency care studies [5, 11].

To test subgroup differences, we applied weighted linear models (equivalent to one-way ANOVA), with population shares used as analytic weights. Pairwise differences between children, reproductive-age women, and older adults were estimated using marginal means and all pairwise contrasts (children vs. women, children vs. elderly, and elderly vs. women) from the emmeans package in R [22]. These demographic groups were analyzed and compared with each other because they correspond to key stages of the life course associated with differing healthcare needs, and are commonly prioritized in health system planning and equity analyses. Statistical significance was defined at *p* < 0.05, with additional reporting for *p* < 0.01 and *p* < 0.001. All analyses were conducted in R version 4.4.0.

## Results

### Geographical Access Across the Urban–Rural Continuum

The average travel time by Degree of Urbanisation levels, weighted by population across grid cells, is summarized in Table 1. Unsurprisingly, cities have the shortest travel times, followed by towns and semi-dense areas, while rural areas consistently experience the longest travel times. Walking travel times are significantly longer than motorized travel times across all urbanization levels, with the difference being most pronounced in rural areas where healthcare facilities are more dispersed. Nigeria generally has shorter travel times than Zambia, particularly in rural areas, where accessing hospitals by foot takes 280 min on average in Nigeria compared to 371 min in Zambia. Similarly, rural residents in Zambia must walk 69 min to reach any health facility, while in Nigeria, the average is 43 min. Motorized transport greatly reduces travel times, but Zambia still experiences longer travel times than Nigeria in urban and rural areas.

**Table 1:** The estimated average travel time (minutes) per capita to the closest health facilities or hospitals by motorized transport and walking, respectively, in Nigeria and Zambia in 2020

Country	Degree of Urbanisation	Any facility		Hospitals	
		Motorized transport	Walking	Motorized transport	Walking
Nigeria	Rural areas	14	43	38	280
	Towns and semi-dense areas	4	16	18	170
	Cities	1	10	4	59
Zambia	Rural areas	17	69	54	371
	Towns and semi-dense areas	3	20	10	86
	Cities	1	13	4	39

Estimates are least-cost, free-flow travel times computed on a 1 km friction surface (aligned with DEGURBA) and should be interpreted as lower bounds, not door-to-door times. City averages can appear very low (≈1 min) because (i) some population pixels coincide with a facility (yielding 0 min), (ii) short source–target distances at 1 km resolution, and (iii) the model omits urban delays (traffic, intersections, parking/boarding, wait times) and strict network constraints. Absolute values—especially for motorized access in cities—are therefore optimistic; the relative differences across settlement classes and between “any facility” and hospitals are more robust

**Fig. 1:**
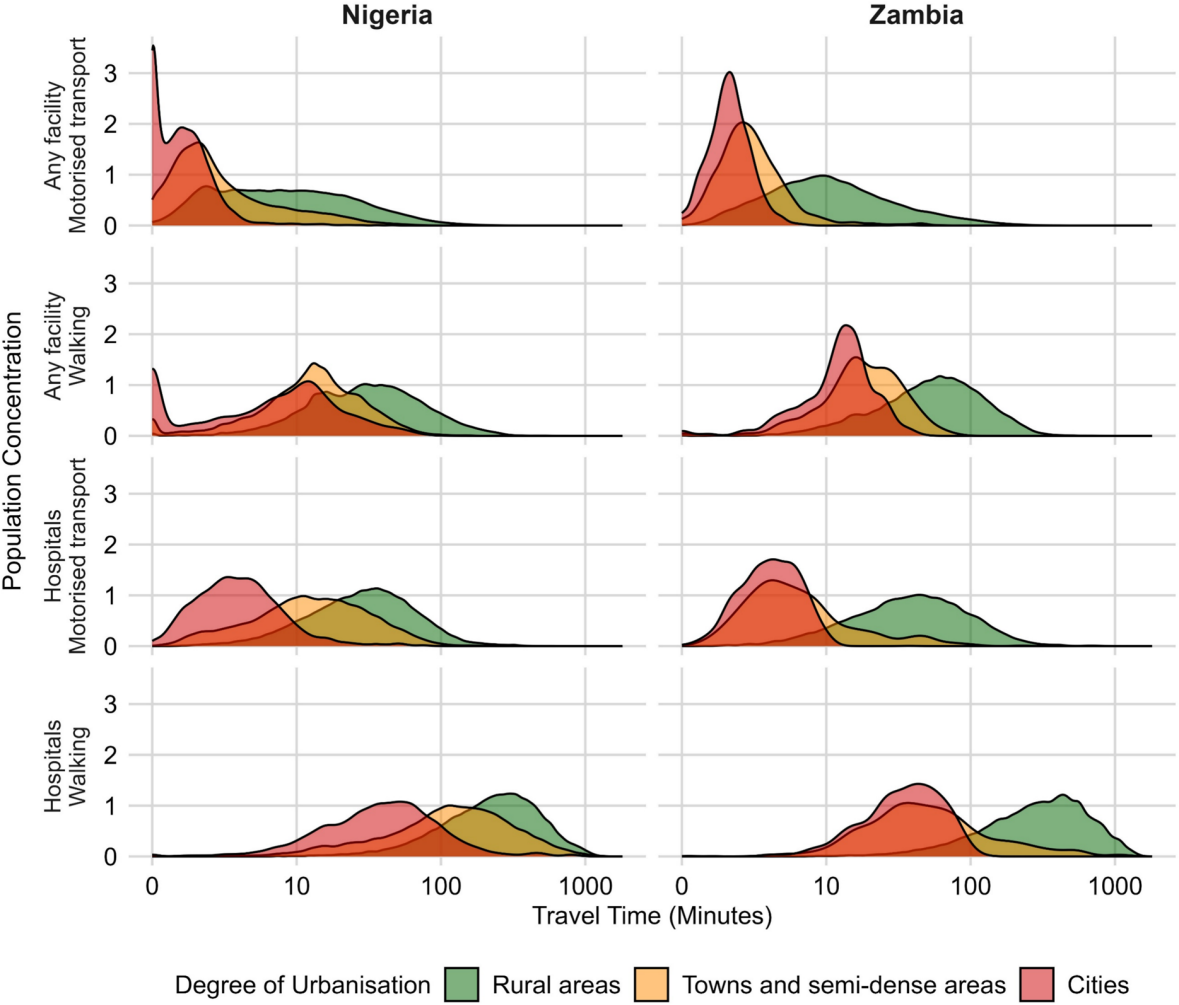
Population-weighted density distributions of travel time to different health facility types in Nigeria and Zambia in 2020 by two transport modes and Degree of Urbanisation. Densities are weighted by total population per grid cell to reflect the relative concentration of population experiencing each travel time

Figure 1 highlights inequality in travel times to health facilities within different urbanization levels by contrasting the width of the distribution of the population concentration for different modes of transport and facility types across countries and urbanization. Cities generally exhibit the narrowest distributions, indicating relatively uniform and shorter travel times within their populations. Rural areas have the widest distribution spread with much longer travel times for their population, highlighting significant intra-area inequality and overall poor accessibility to medical services. Some rural residents even have much shorter travel times than some urban populations to access medical services in both Nigeria and Zambia. The disparities are clearer for walking access to hospitals, where rural populations experience the greatest variation and longest travel durations.

### Geographic Accessibility Coverage Across the Urban–Rural Continuum

**Fig. 2:**
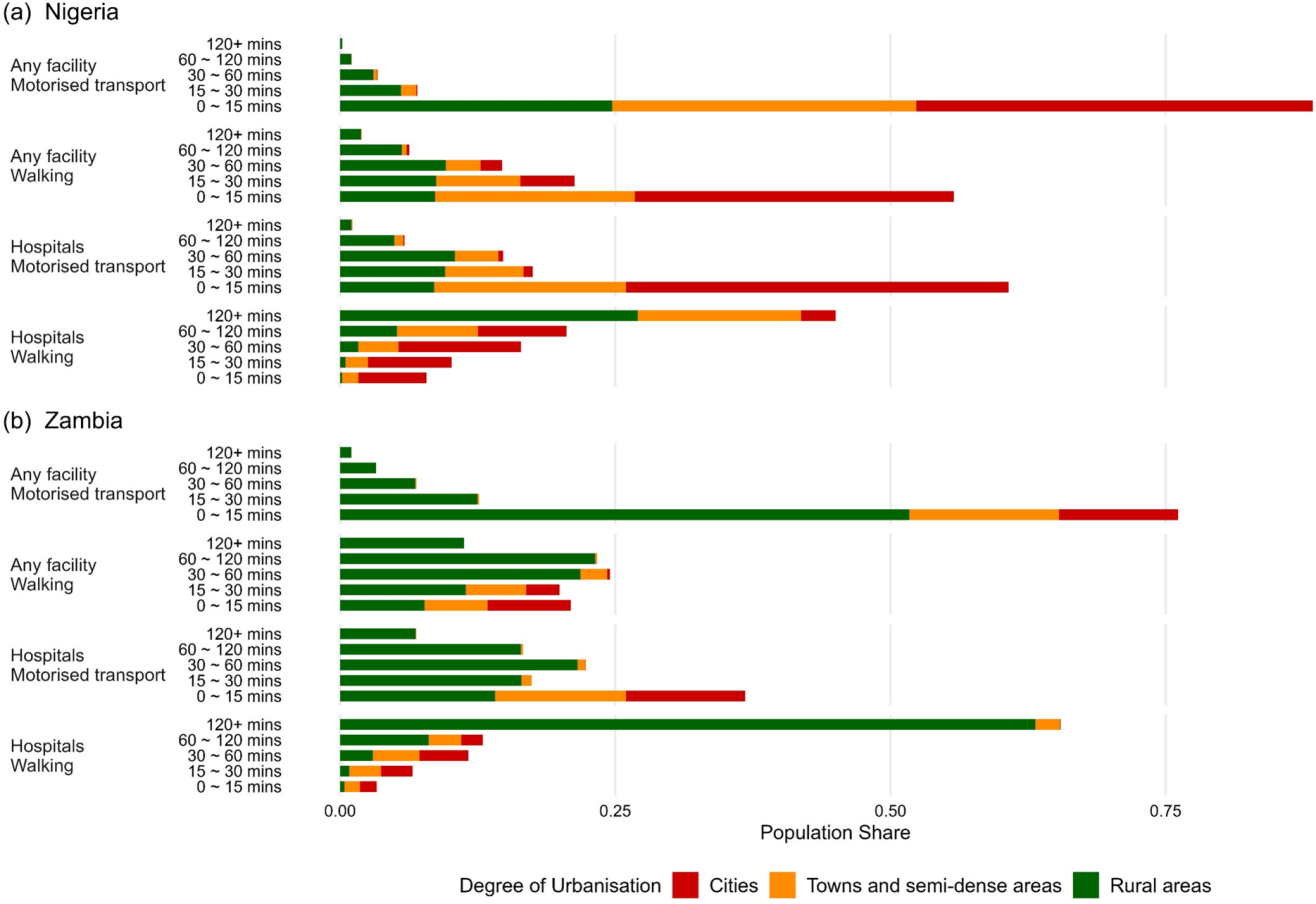
Estimated geographic accessibility coverage by health facility types, transport modes, and Degree of Urbanisation for Nigeria and Zambia in 2020. Population share refers to the percentage of the total population within each country and transport mode that falls into a given travel-time category, with shares summing to 100% across all categories for each country–mode combination

The estimated geographic accessibility of healthcare services varies significantly in terms of population shares across travel-time categories and transport modes, as shown in Fig. 2. Here, population share refers to the percentage of the total population within each country and transport mode that falls into a given travel-time category, with shares summing to 100% across all categories for each country–mode combination. For example, a value of 56% in the 0–15 min walking category means that 56% of the national population can reach a health facility within 15 min on foot, with the remainder experiencing longer travel times.

In Nigeria, a large proportion of the population benefits from short travel times, with 88% of the total population able to reach the closest any facility within 15 min by motorized transport, while 61% can reach hospitals within the same timeframe. Walking geographic accessibility is estimated to be more limited, with 56% of the population able to reach the closest health facility within 15 min, and this percentage drops to 8% for hospitals. Zambia shows a similar pattern, with 77% of the total population able to reach any facility within 15 min by motorized transport, while 44% can reach hospitals within the same timeframe. Nonetheless, only 31% of the population (only half compared to Nigeria) is able to reach the closest facility of any type within 15 min by walking, and this percentage drops to 6% for hospitals.

In Nigeria in 2020, as travel time increases, estimated population shares gradually decline whereas the trend is reversed for walking access to hospitals. For example, for motorized access to hospitals in Nigeria, 17% of the population falls within the 15–30-min range, 15% within 30–60 min, and 6% within 60–120 min. While walking access to hospitals shows 10% of the population falls within the 15–30-min range, 16% within 30–60 min, and 21% within 60–120 min catchments. It indicates a larger proportion of people face longer travel times if transport is unavailable, with notably 45% of the population requiring over 120 min to reach hospitals on foot, illustrating critical access challenges for those without motorized transport. Zambia has similar patterns, while the population shares are relatively stable for estimated walking access to the closest health facility, where 20% of the population falls within the 15–30-min range, 25% within 30–60 min, and 23% within 60–120 min catchments. In addition, compared to Nigeria, a larger portion of the population in Zambia faces severe healthcare geographic accessibility challenges, with 11% (2% in Nigeria) of the population needing more than 120 min walking to the closest health facility, 7% (1% in Nigeria) of the population for motorized transport to hospitals, and 65% (45% in Nigeria) by walking to hospitals.

Figure 2 also reflects inequality of geographic access to health facilities across Degree of Urbanisation levels under fixed travel time. In the shortest travel-time catchments (0–15 min), Nigeria’s urban populations dominate, representing the largest share (40–75% in terms of different modes of transport), followed closely by towns and semi-dense areas. While the rural population in Zambia usually takes a larger share within this catchment, except for walking access to hospitals where still only a small portion of the rural population can walk to a hospital within 15 min. More than 50% of the population having access to the closest health facility of any type within 15 min via motorized transport are from Zambia’s rural area, suggesting that healthcare access appears more evenly distributed in Zambia compared to Nigeria. Nonetheless, the rural population consistently makes up the overwhelming majority of those facing extreme healthcare inaccessibility, i.e., beyond the 120-min threshold. Nearly all individuals requiring over 120 min to reach either a hospital or a health facility are from rural areas, with the only exception being walking-based hospital access. Even in this case, the rural population still accounts for a significantly larger share compared to other scenarios. This reveals a clear disparity in healthcare provision across the urban–rural continuum, where some rural residents are disproportionately left without timely medical access, further emphasizing the urgent need for targeted interventions to improve geographic healthcare accessibility in these underserved areas.

### Breakdown by Age and Sex Groups

**Fig. 3:**
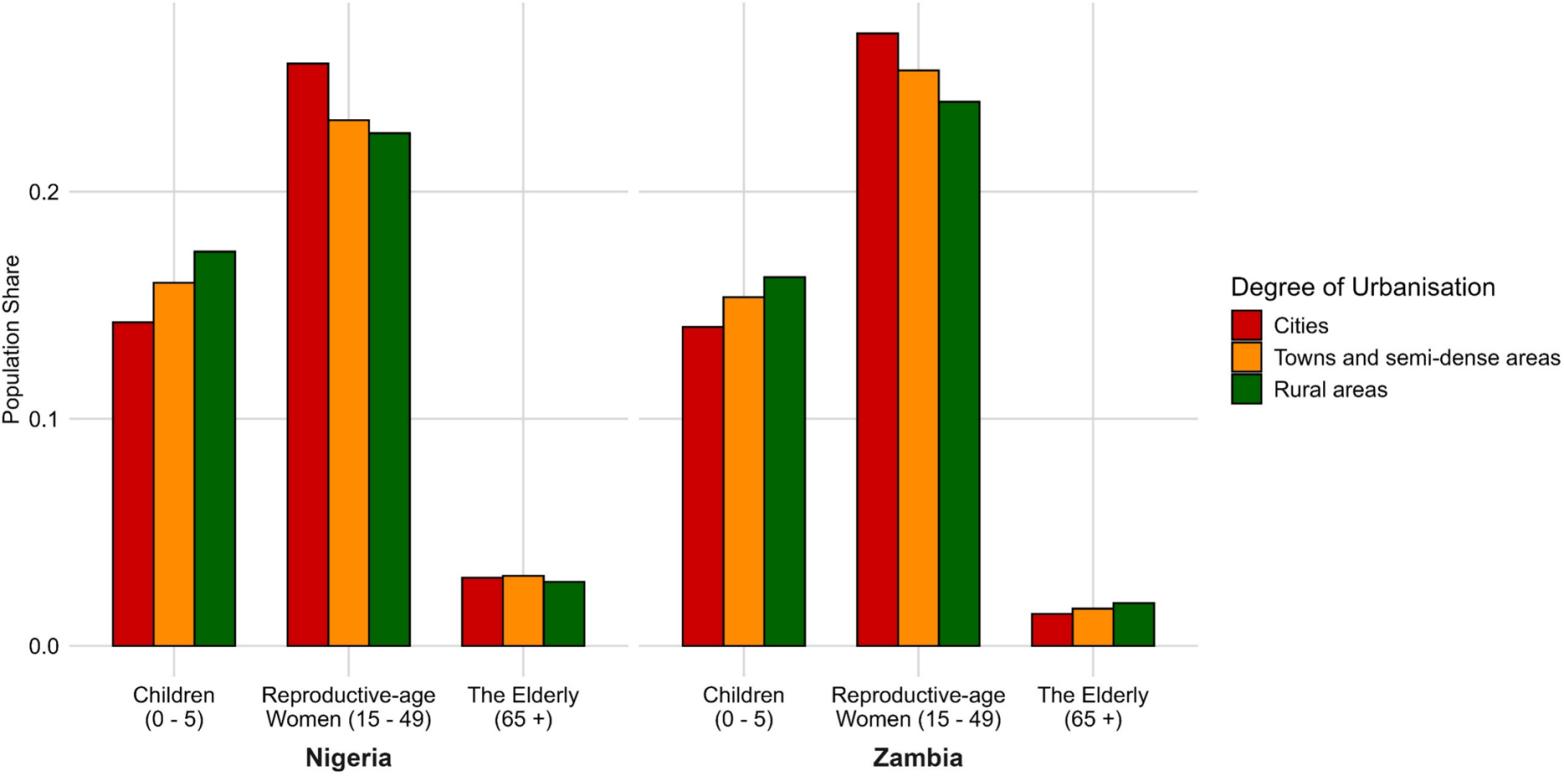
Estimated population shares by age and sex groups and Degree of Urbanisation in Nigeria and Zambia in 2020. Percentages represent the proportion of the total national population in each country belonging to a given demographic group (children aged 0–5, reproductive-age women aged 15–49, and older adults aged 65 +) within each Degree of Urbanisation category

Figure 3 illustrates the estimated distribution of children (0–4 years old), reproductive-age women (15–49 years old), and the elderly (65 + years old) population across different Degrees of Urbanization levels in Nigeria and Zambia in 2020, respectively. Percentages represent the share of the total national population in each country belonging to each demographic group within a given Degree of Urbanisation category. Both countries exhibit relatively high child population shares in rural areas, 17.36% in Nigeria and 16.24% in Zambia, reflecting the younger populations in these areas. The child population share declines as urbanization increases, dropping to 15.98% in Nigeria and 15.36% in Zambia in towns and semi-dense areas, and further reductions to 14.25% in Nigeria and 14.04% in Zambia in cities. Reproductive-age women show a reverse pattern. In Nigeria, the reproductive-age women share increases from 22.58% in rural areas to 23.15% in towns and semi-dense areas, and to 25.64% in cities. And in Zambia from 23.96% in rural areas, to 25.34% in towns and semi-dense areas, and to 26.97% in cities. The elderly population shares show slight differences across the urban–rural continuum, but the trends are different in Nigeria and Zambia. Nigeria records an estimated 2.82% in rural areas, which increase to 3.09% in towns and semi-dense areas, and remaining relatively stable at 3.00% in cities. Zambia has lower estimated elderly population shares, with 1.88% in rural areas, dropping slightly to 1.64% in towns and semi-dense areas, and a further reduction to 1.41% in cities.

Table 2 documents subgroup differences of travel time to health facilities by Degree of Urbanisation. In Nigeria, children consistently face significantly longer travel times than reproductive-age women and the elderly. For example, in rural areas, children are estimated to walk 23.27 min longer on average than the elderly and 15.77 min longer than reproductive-age women to reach a hospital. In contrast, Zambia shows fewer and less consistent differences between groups, with occasional disadvantages for elderly people, particularly in motorized access scenarios, but most comparisons remain statistically non-significant. In summary, while most group differences are small and non-significant, especially in urban areas and when motorized transport is available, specific group vulnerabilities emerge under less favorable conditions. Children in Nigeria are particularly disadvantaged in rural settings when walking is required, pointing to the need for targeted support in these environments.

**Table 2:** Pairwise estimated travel time differences (in minutes) for 2020 across demographic groups within the same Degree of Urbanisation

Degree of Urbanisation	Healthcare types	Group comparison	Motorized		Walking	
			Nigeria	Zambia	Nigeria	Zambia
Rural areas	Any facility	C – E	1.08***	− 0.45**	3.14***	− 0.44
		C – W	0.66***	0.86***	1.57***	1.73***
		E—W	− 0.42***	1.31***	− 1.58***	2.17***
	Hospitals	C – E	2.69***	− 1.14***	23.27***	− 4.24**
		C – W	1.68***	3.02***	15.77***	15.70***
		E—W	− 1.01***	4.16***	− 7.51***	19.94***
Towns and semi-dense areas	Any facility	C – E	0.58***	0.05	1.15***	0.49
		C – W	0.41***	0.11	0.69***	0.06
		E—W	− 0.17*	0.05	− 0.46**	− 0.43
	Hospitals	C – E	2.71***	0.26	26.07***	4.50
		C – W	1.98***	0.80	19.06***	3.33
		E—W	− 0.73***	0.55	− 7.01***	− 1.17
Cities	Any facility	C – E	0.02	0.02	0.26	0.25
		C – W	0.00	0.02	− 0.03	0.03
		E—W	− 0.02	0.00	− 0.28	− 0.23
	Hospitals	C – E	0.46**	0.03	6.82***	0.37
		C – W	0.29***	0.04	3.79***	0.26
		E—W	− 0.17	0.01	− 3.03	− 0.11

All values represent pairwise differences between the two demographic groups listed. Positive values indicate the first group listed had longer travel times than the second; negative values indicate shorter travel times

*C* children, *W* reproductive-age women, *E* the elderly people

Significance is assessed using the weighted ANOVA with the following thresholds: *p < 0.05, **p < 0.01, and ***p < 0.001

## Discussion

This study provides new evidence on geographic inequalities in geographic healthcare accessibility in Nigeria and Zambia by applying the UN-endorsed Degree of Urbanisation framework. Our findings highlight that access to healthcare is strongly structured by the urban–rural continuum, where rural populations consistently face the longest travel times, with walking-based access to hospitals often exceeding 4 h. Besides, transport availability is a critical determinant of access, with motorized travel dramatically reducing travel times, but leaving those reliant on walking severely disadvantaged. More importantly, disparities are not uniform across demographic groups. In Nigeria, children in rural areas face significantly longer hospital travel times than reproductive-age women and older adults, while in Zambia, subgroup differences are less pronounced. Together, these findings underscore the importance of considering both geographic and demographic dimensions when designing policies to achieve universal health coverage (UHC).

Our results are consistent with a large body of literature demonstrating persistent urban–rural inequalities in geographic healthcare accessibility across LMICs. Previous research has shown that rural residents often experience longer travel times and limited-service provision, contributing to worse maternal, child, and general health outcomes [5, 9]. In both Nigeria and Zambia, rural disadvantage was particularly acute for walking access, aligning with global evidence that distance and transport costs remain key barriers to healthcare in low-resource settings [6, 7]. These results also support studies highlighting the importance of transport infrastructure: where road networks are sparse or poorly maintained, health facility coverage may be insufficient to guarantee timely access [23].

At the same time, our findings highlight significant intra-area inequality within rural regions. Even neighboring communities may experience starkly different levels of access due to variations in physical geography, road connectivity, and infrastructure investment [24, 25]. Some rural settlements, especially those near urban fringes, benefit from spillover services and infrastructure, while others remain extremely isolated. These patterns exacerbate existing socio-economic vulnerabilities, as poorer rural communities not only face longer travel times but also have fewer resources to overcome these barriers [26].

The demographic analysis provides further nuance. In both Nigeria and Zambia, children constitute a larger share of rural populations, while reproductive-age women are more concentrated in towns and cities, reflecting established fertility and migration dynamics [27]. Our results indicate that children in Nigeria face systematically longer travel times to hospitals compared to adults, particularly in rural areas. This disparity suggests that pediatric care is insufficiently available in remote communities, a finding consistent with studies documenting poorer child health outcomes in rural sub-Saharan Africa [5]. Targeted solutions, such as mobile health clinics for child and maternal health, as well as subsidized transportation for emergencies, could help reduce these inequities.

The use of the Degree of Urbanisation (DEGURBA) framework offers clear advantages for health equity research. Unlike administrative boundaries or national definitions of “urban” and “rural,” DEGURBA provides a harmonized and globally comparable classification of settlements [15, 16]. This makes it possible to assess disparities across the urban–rural continuum in a consistent way, supporting international comparisons and SDG monitoring [17]. By linking this classification with population and facility data, our study highlights how harmonized geographic frameworks can help policymakers more accurately identify underserved populations and allocate resources effectively.

From a policy perspective, our findings stress the need for multi-pronged strategies to address geographic inequities in healthcare access. For rural areas, investments in transport infrastructure are essential, as walking times are prohibitively long and motorized transport remains limited. These findings echo broader evidence on how transport systems, commuting times, and mass transit infrastructure interact with health outcomes in urban environments [28–30]. Expanding and maintaining rural road networks, combined with targeted health facility placement, could substantially reduce disparities [23]. In parallel, community- and mobile-based health services may offer interim solutions for hard-to-reach populations, particularly for children in Nigeria who face the greatest disadvantages [31]. Urban health systems should also not be neglected. Although average travel times in cities were short, intra-urban disparities persist, especially in peri-urban settlements with limited infrastructure.

We acknowledge several limitations in our study. First, we modeled access at 1 km resolution to align with DEGURBA classification, which provides consistency across countries but may underestimate intra-urban travel times due to the smoothing of road networks. Other studies using finer-scale street networks report more realistic urban estimates [32]. Second, travel times were estimated as least-cost, free-flow conditions, and therefore do not account for delays such as traffic congestion, boarding times, or healthcare facility quality. Third, our analysis relies on geolocated health facility datasets that, while among the most comprehensive available nationally, may not capture all facilities, particularly informal providers, very small private clinics, or recently established facilities in remote rural areas. Such omissions could lead to overestimation of travel times in some locations. Fourth, demographic subgroup estimates rely on gridded population datasets that disaggregate census proportions at the administrative-unit level. As a result, within-unit variation in subgroup distributions may be underestimated. Finally, different gridded population choices may impact the modeled estimates in terms of both the DEGURBA classification [33] and the assessment of geographic access to healthcare [34].

Despite these limitations, this study demonstrates the value of harmonized spatial frameworks for understanding geographic healthcare accessibility. By applying DEGURBA to Nigeria and Zambia, we identified geographic disparities and highlighted demographic groups, particularly rural children in Nigeria, at greatest risk of being left behind. Addressing these inequities will require both structural investments in health systems and transport networks, as well as targeted policies for vulnerable populations. More broadly, integrating harmonized geographic methods into health system research and planning can support progress toward universal health coverage, ensuring that services are equitably distributed across the full urban–rural continuum and even across countries globally.

## Conclusion

This study demonstrates that geographic access to healthcare in Nigeria and Zambia is deeply unequal, with rural residents, particularly children, facing the greatest barriers to timely care. While urban populations benefit from dense service networks and shorter travel times, our results show that motorized transport remains critical to overcoming distance in rural areas, where reliance on walking leaves large segments of the population underserved. Importantly, these disparities are not uniform, i.e., even within rural settings, proximity to infrastructure or transport networks creates significant inequalities between neighboring communities.

By applying the Degree of Urbanisation framework, we provide a consistent way to compare access across settlement types and demographic groups, revealing both cross-country differences and vulnerable populations that require targeted interventions. For policymakers, the findings stress the importance of strengthening rural transport systems, investing in strategically located health facilities, and considering mobile or community-based services to meet the needs of remote populations. Addressing these persistent gaps is essential to achieving universal health coverage and ensuring that health systems respond equitably to the diverse realities of urban, peri-urban, and rural populations.

## Data Availability

DEGURBA level 1 datasets (Zambia, Nigeria): 10.13130/RD_UNIMI/YFNFZC; WorldPop Global2 age–sexgridded population: https://hub.worldpop.org/geodata/listing?id=136; GRID3 Nigeria health facilities v2.0: 10.7916/kv1n-0743; GRID3 Zambia operational health facilities (v01): 10.7916/d8-xksb-0t02.
